# Hemoglobin α and β are ubiquitous in the human lung, decline in idiopathic pulmonary fibrosis but not in COPD

**DOI:** 10.1186/1465-9921-11-123

**Published:** 2010-09-13

**Authors:** Nobuhisa Ishikawa, Steffen Ohlmeier, Kaisa Salmenkivi, Marjukka Myllärniemi, Irfan Rahman, Witold Mazur, Vuokko L Kinnula

**Affiliations:** 1 Department of Medicine, Pulmonary Division, P.O. Box 22 (Haartmaninkatu 4), FI-00014 University of Helsinki and Helsinki University Central Hospital, Helsinki, Finland; 2 Department of Molecular and Internal Medicine, Graduate School of Biomedical Sciences, Hiroshima University, 1-2-3 Kasumi, Minami-ku, Hiroshima 734-8551, Japan; 3 Proteomics Core Facility, Biocenter Oulu, Department of Biochemistry, University of Oulu, Linnanmaa, P.O. Box 3000, FI-90014 Oulu, Finland; 4 Departments of Virology and Pathology, Haartman Institute, P.O. Box 21, FI-00014 University of Helsinki, Helsinki, Finland; 5 Department of Environmental Medicine, Lung Biology and Disease Program, University of Rochester Medical Center, Box 850, 601 Elmwood Avenue, Rochester, NY 14642, USA

## Abstract

**Background:**

Idiopathic pulmonary fibrosis (IPF) and chronic obstructive pulmonary disease (COPD) are disorders of the lung parenchyma. They share the common denominators of a progressive nature and poor prognosis. The goal was to use non-biased proteomics to discover new markers for these diseases.

**Methods:**

Proteomics of fibrotic vs. control lung tissue suggested decreased levels of several spots in the lung specimens of IPF patients, which were identified as Hemoglobin (Hb) α and β monomers and Hbα complexes. The Hbα and β monomers and complexes were investigated in more detail in normal lung and lung specimens of patients with IPF and COPD by immunohistochemistry, morphometry and mass spectrometry (MS).

**Results:**

Both Hb monomers, in normal lung, were expressed especially in the alveolar epithelium. Levels of Hbα and β monomers and complexes were reduced/lost in IPF but not in the COPD lungs when compared to control lung. MS-analyses revealed Hbα modification at cysteine105 (Cysα105), preventing formation of the Hbα complexes in the IPF lungs. Hbα and Hbβ were expressed as complexes and monomers in the lung tissues, but were secreted into the bronchoalveolar lavage fluid and/or induced sputum supernatants as complexes corresponding to the molecular weight of the Hb tetramer.

**Conclusions:**

The abundant expression of the oxygen carrier molecule Hb in the normal lung epithelium and its decline in IPF lung are new findings. The loss of Hb complex formation in IPF warrants further studies and may be considered as a disease-specific modification.

## Background

Idiopathic pulmonary fibrosis (IPF) (histopathology of usual interstitial pneumonia, UIP) is classified as one of the idiopathic interstitial pneumonias, representing an entity with unknown etiology, aggressive fibrogenesis and a very poor prognosis [1,2]. IPF is considered primarily as a disease associated with epithelial/fibroblastic pathology [3,4]. Chronic obstructive pulmonary disease (COPD) is a slowly progressive but very common lung disease, with most of the cases being related to smoking. COPD involves not only airway inflammation/obstruction but also varying degrees of parenchymal lung damage i.e. emphysema combined with small airway fibrosis and the occurrence of patchy fibrotic lesions in the lung parenchyma. Despite recent advances in our understanding of the pathogenesis of these diseases, the precise molecular mechanisms leading to their progression remain unclear, and there is no effective therapeutic strategy for either of these disorders.

Both IPF and COPD have been shown to be associated with oxidative/nitrosative stress [5-7]. The elevated oxidant burden in turn triggers the activation of growth factors and metalloproteases and evokes an imbalance in the acetylases/deacetylases and disruptions of the transcription of several inflammatory genes in the lung [8,9]. Due to several overlapping features between chronic airway and parenchymal lung diseases, there is an urgent need to understand better disease specific changes in order to pinpoint their exact diagnosis and response to treatment.

The present study was undertaken to use non-biased proteomics to clarify the mechanisms related to these two lung diseases i.e. IPF and COPD, and to identify disease specific markers. Our recent proteomic approaches at pH 4-7 have revealed altered expression of several spots in the lung specimens of COPD and IPF, which were identified to represent surfactant protein A [10] and various RAGE (receptor for advanced glycation endproducts) isoforms [11]. Further screening at pH 6-11 revealed a loss of a third group of proteins in the lung specimens of the patients with IPF; corresponding changes could not be found in the COPD lung. These spots were identified by MS and found to represent Hemoglobin (Hb) α and β monomers and Hbα complexes. The wide spectrum of Hb functions extends from oxygen (O_2_) binding and transport, nitric oxide (NO) metabolism, blood pressure regulation, to protection against oxidative and nitrosative stress [12-14]. The distribution, expression or significance of Hb and its subchains have not been investigated in lung diseases. In this study, Hbα and Hbβ monomers and complexes were investigated in more detail in normal lung and lung specimens of patients with IPF and COPD by Western blot, immunohistochemistry, morphometry and mass spectrometry (MS). In addition, Hb (α, β) levels in bronchoalveolar lavage (BAL) and induced sputum samples were investigated to elucidate whether Hb would be detectable in these samples and could possibly be used in the evaluation of these diseases.

## Methods

### Study subjects

Tissue samples were collected by lung surgery from patients treated in Helsinki University Central Hospital. All control tissues were obtained from lung surgery from hamartomas or from the surgery of local tumors (controls), or from lung transplantations (COPD Stage IV and IPF lung). Bronchoalveolar lavage fluid (BALF) and sputum samples were collected from patients of the Division of Pulmonary Medicine, Helsinki University Central Hospital or healthy volunteers. Each IPF case was confirmed and re-evaluated to represent UIP histopathology by an experienced pathologist. COPD was defined according to GOLD criteria (FEV1 < 80% of predicted, FEV1/FVC < 70% and bronchodilatation effect < 12%) [15,16]. Five to 10 mg oral predonisolone and/or inhaled corticosteroids had been included in the regular therapy of all IPF patients and Stage IV (very severe) COPD, none of the other subjects were receiving regular corticosteroid therapy. The Ethics Committee of the Helsinki University Central Hospital approved the study and all patients received written information and gave their permission to use the samples. Characteristics of the patients are shown in Tables 1, 2 and 3.

**Table 1:** Characteristics of the controls, IPF and COPD patients in the 2-DE analyses of the lung homogenates

	Control	COPD Stage IV	IPF
Patients, n	4	4	4
Age, yr	59 ± 7	58 ± 4	54 ± 5
Sex, M/F	3/1	1/3	3/1
Pack years, yr	< 12 *	32 ± 2***	15**
FEV1 (%)	89 ± 10	12 ± 2***	38 ± 3***
FVC (%)	77 ± 1	31 ± 4 ***	35 ± 3***

Data are presented as mean ± SEM.

* Three of the controls were never smokers and one of the controls had smoked 10 to 12 years but stopped smoking at least 2 years before the study.

** Three of the IPF patients were never smokers and one of the IPF patients had smoked but stopped smoking 30 years before the surgery.

*** p < 0.05 versus control subjects. All patients with COPD stage IV had been smoked but stopped smoking at least 2 years before the study.

**Table 2:** Characteristics of the control, COPD and IPF patients in the Hemoglobin alpha and beta Western blot analyses of the lung homogenates

	Control	Smoker	COPD Stage IV	IPF
Patients, n	7	7	7	7
Age, yr	65 ± 3	62 ± 3	58 ± 2	56 ± 3
Sex, M/F	4/3	6/1	4/3	5/2
Pack years, yr	7 ± 5 *	21 ± 6	31 ± 5**	6 ± 5 ***
FEV1 (%)	100 ± 6	88 ± 3	22 ± 5^#^	47 ± 6^#^
FVC (%)	102 ± 6	87 ± 4	47 ± 8^#^	43 ± 5^#^

Data are presented as mean ± SEM.

* Four of the controls were never smokers and two of the controls had smoked 10-15 years but stopped smoking at least 2 years before the study; one of the controls had smoked 30 years but stopped smoking one year before the study.

** All the patients were smokers, but had stopped smoking at least 2 years before the study. *** Five of the IPF patients were never smokers; two had smoked for over 5 years but stopped smoking at least two years before the study.

^# ^p < 0.05 versus control subjects and healthy smokers.

**Table 3:** Characteristics of the controls, COPD and IPF patients in the immunohistochemical analyses of the lung

	Control	COPD Stage IV	IPF
Patients, n	6	7	7
Age, yr	64 ± 3	60 ± 2	61 ± 3
Sex, M/F	5/1	4/3	7/0
Pack years, yr	10 ± 7 *	35 ± 5**	15 ± 9 ***
FEV1 (%)	105 ± 5	40 ± 9 ****	60 ± 8****
FVC (%)	104 ± 6	59 ± 6****	57 ± 7****

Data are presented as mean ± SEM.

* Two of the controls were never smokers and four of the controls had smoked 10-30 years but stopped smoking at least 2 years before study.

** The patients were ex-smokers, but had stopped smoking at least 2 years before the study.

*** Three of the IPF patients were never smokers, four had smoked 15-45 years but stopped smoking at least two years before the study.

**** p < 0.05 versus control subjects.

### Bronchoalveolar lavage fluid (BALF)

Bronchoalveolar lavage was performed under local anesthesia to a representative lung segment with 200 ml of sterile 0.9% saline according to the standard procedure as described [17]. The fluid was centrifuged at 400 ×*g *for 10 min at +4°C to separate the cells from the supernatant. The supernatants were divided into smaller aliquots and stored at -80°C for further experiments. The subjects represented patients who had been investigated for prolonged cough, but whose lung function, high resolution computed tomography (HRCT) and BAL cell profiles were normal and who recovered spontaneously with no specific diagnosis for any lung disease. Characteristics of the patients are shown in Table 4.

**Table 4:** Characteristics of the control subjects in the Hemoglobin Western blot analyses from the BAL fluid and sputum supernatant

	Control (Prolonged cough) BALF *	Control (Non-smokers) Sputum
n	6	7
Age, yr	43 ± 9	50 ± 5
Sex, M/F	2/4	6/1
Smoking/non-smoking	6/1 **	0/7
FEV1	3.5 ± 0.5	4.1 ± 0.25
FVC	4.3 ± 0.6	5 ± 0.4

Data are presented as mean ± SEM.

* Subjects with prolonged cough without any interstitial or alveolar abnormalities by high-resolution computed tomography (HRCT) and with a normal cell profile in the BALF.

### Induced sputum

Sputum was induced by inhalation of hypertonic saline as recommended by the European

Respiratory Society Task Force and processed as described [18,19]. Induced sputum supernatants for Western blot were collected and immediately transferred to -80°C. The specimens were obtained from healthy nonsmokers whose lung function values were normal. Characteristics of these subjects are shown in Table 4.

### Two-Dimensional Gel Electrophoresis (2-DE) and Protein Identification

2-DE analyses were performed as described earlier [10,11]. Frozen lung tissue samples were powdered and further purified by acetone precipitation. The protein extract was resuspended in urea buffer (6 M urea, 2 M thiourea, 2% [w/v] CHAPS, 0.15% [w/v] DTT, 0.5% [v/v] carrier ampholytes 3-10, Complete Mini protease inhibitor cocktail [Roche]), incubated for 10 minutes in an ultrasonic bath, and centrifuged. Protein aliquots (100 μg) were stored at -20°C. In the alkylation experiment, the protein extract in alkylation buffer containing 6 M urea, 2% [w/v] CHAPS, 65 mM DTT and Complete Mini protease inhibitor cocktail was incubated for 15 min at RT with 130 mM iodoacetamide. The protein separation for each sample (control lung, IPF and Stage IV COPD) was done in triplicate. IPG, strips (pH 6-11, 18 cm, GE Healthcare) were rehydrated in 350 μl urea buffer overnight. Prior to application into sample cups at the anodic end of the IPG, the protein solution was adjusted with urea buffer to a final volume of 100 μl. Isoelectric focusing (IEF) was carried out with the Multiphor II system (GE Healthcare) under paraffin oil for 85 kVh. SDS-PAGE was performed overnight in polyacrylamide gels (12.5% T, 2.6% C) with the Ettan DALT II system (GE Healthcare) at 1-2 W per gel and 12°C. The total protein in the gel was visualized by silver staining. The protein pattern was analyzed with the 2-D PAGE image analysis software Melanie 3.0 (GeneBio). The exact positions (isoelectric point [pI], molecular mass) of the spots were determined from the reference 2-D gel of human lung (pH 6-11) with the identified marker proteins. The expected spot position was calculated with the Compute pI/Mw tool (http://au.expasy.org/tools/pi_tool.html).

In the protein identification, excised spots were digested as described [11]. Peptide masses were measured with a VOYAGER-DE™ STR [11] and proteins identified by full database search (Aldente database version 11/02/2008 (http://ca.expasy.org/tools/aldente/) according to the following parameters (20 ppm; 1 missed cut; [M+H]; +CAM; +MSO). Further information about the proteins was obtained from the Swiss-Prot/TrEMBL database (http://au.expasy.org/sprot/) and NCBI database (http://www.ncbi.nlm.nih.gov/).

### Western Blot Analysis

Western blot analyses of lung tissue homogenates were performed as described [20-22]. Tissue samples were homogenized in PBS, and 50 μg of protein was used under standard i.e. reducing or non-reducing conditions [23]. Membranes were probed with goat anti-Hemoglobin alpha (Hbα) antibody (H80: sc-21005, Santa Cruz Biotechnology, Inc. Santa Cruz, CA) or mouse anti-Hemoglobin beta (Hbβ) antibody (M02, Abnova, Taipei, Taiwan), followed by secondary antibody treatments. Since the expressions of housekeeping proteins (e.g. β-actin but possibly also others) vary in airway and parenchymal lung diseases including COPD [10,11], equal loading was standardized against Ponceau S staining of the membranes (Sigma Aldrich, St. Louis, MO) [24-26]. Quantitative analysis of the Western blot bands as well as the calculation of the corresponding molecular masses was done with Image J 7.0 software (National Institutes of Health, Bethesda, MD).

### Immunohistochemistry and morphometry

Four mm thick paraffin-embedded tissue sections were deparaffinized in xylene and rehydrated in graded alcohol. NovoLink polymer detection system (RE7150-CE, Novocastra Laboratories ltd, Newcastle Upon Tyne, UK) was used for immunostaining according to the manufacturer's instructions. In order to determine the specificity of the staining series, negative control sections were treated with mouse isotype control (Zymed Laboratories, San Francisco, CA, USA) or PBS. Detailed localization of the expression was further investigated using a large magnification (900×). Digital morphometry of the stained tissue sections was conducted as described [27]. Two or three representative images from the lung parenchyma of each stained section were taken with an Olympus U-CMAD3 camera (Olympus Corporation, Japan) and QuickPHOTO CAMERA 2.1 software (Promicra, Prague, Czech Republic). The areas of positively vs negatively stained interstitium or alveolar epithelium were measured with Image-Pro Plus 6.1. software (Media Cybernetics, UK).

### Oxidative/Nitrosative Stress

Nitrotyrosine was used as a marker for oxidative/nitrosative stress because it reflects both superoxide and nitric oxide-mediated reactions in the cells [28]. Nitrotyrosine distribution and expression in the lung sections of control, IPF and COPD lung were assessed by immunohistochemistry, as described [22,29]. Detection of nitrotyrosine was performed with a rabbit anti-nitrotyrosine antibody (06-284, Upstate).

### Statistical Analysis

Data are presented as mean ± SEM. SPSS for Windows (Chicago, IL) was used for statistical analysis and the significance of the associations between two and more than two variables was assessed with Mann-Whitney U and Kruskal Wallis test, respectively. Data was calculated as mean from at least two concurrent samples of several tissue sections of IPF and control; p ≤ 0.05 was considered statistically significant.

## Results

### Loss of Hbα in the IPF but not in the COPD lungs

**Figure 1:**
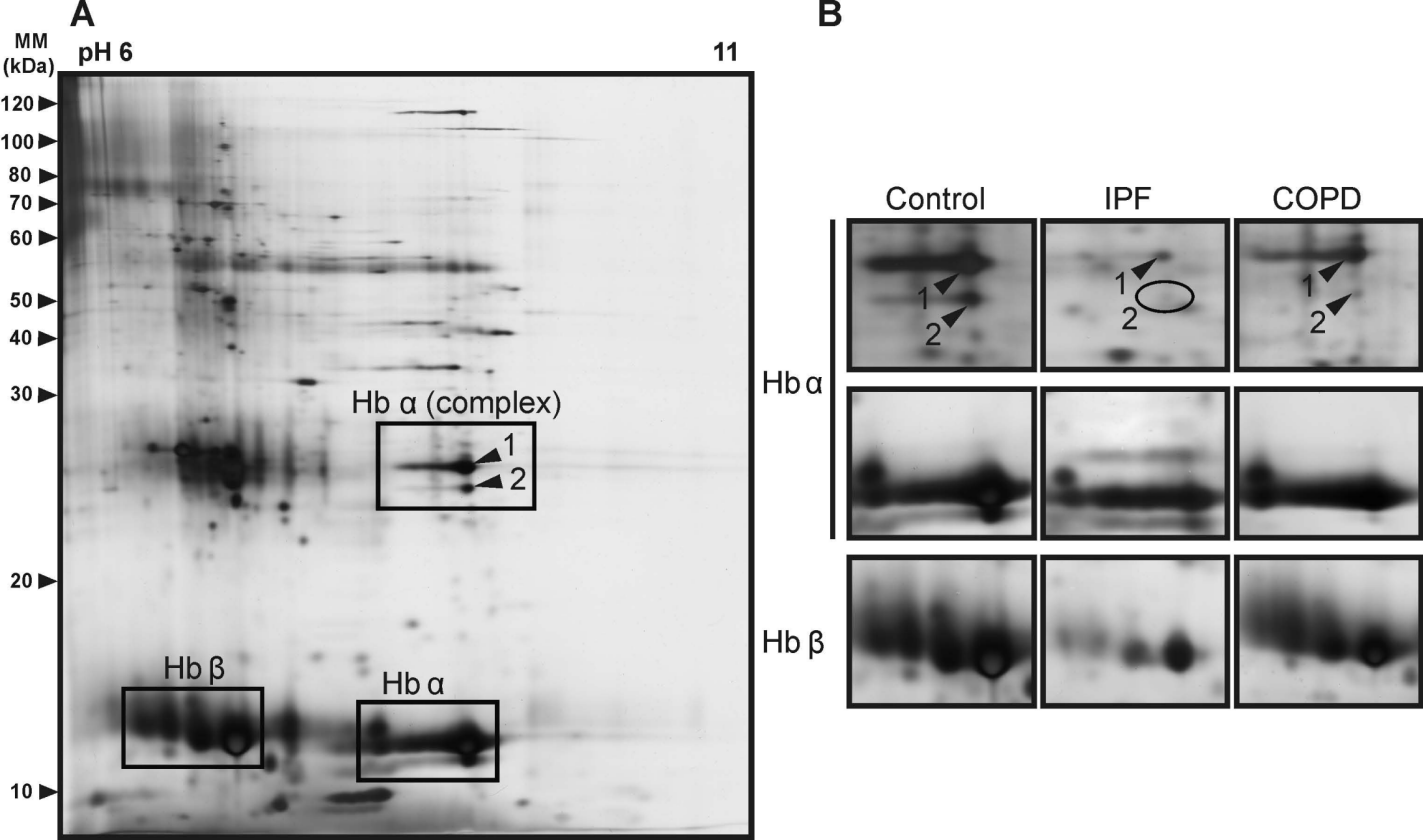
**Two-dimensional gel electrophoresis (2-DE) reveals alterations of Hbα and β monomers and Hbα complexes in IPF lungs**. (A) A representative 2-D gel for control lung is shown on the left. The enlarged gel positions (B) represent Hbα and β monomers and Hbα complexes (spots 1 and 2) in human control (n = 4), IPF (n = 4) and COPD (Stage IV, n = 4) lung tissue. Lung homogenates were separated by 2-DE (pH6-11) and the protein pattern visualized by silver staining. For patient characteristics see Table 1.

Homogenates from control (n = 4) and IPF (n = 4) lung tissues were separated by 2-DE at pH6-11 to search for IPF -specific markers. Two highly abundant "spot trains" at 15 kDa and two spots at a higher molecular mass in the 2-D gels of all control lungs could be detected, which were absent or considerably reduced in the IPF lungs (Figure 1). The comparison with COPD (Stage IV, n = 4), indicated that these alterations were specific for IPF i.e. no evidence for changes in these spots could be seen in the COPD lungs. MS analyses revealed that the "spot trains" represented Hbα and Hbβ whereas Hbα was also identified in the other spots. The position of both "spot trains" in the 2-D gel and the theoretical molecular masses of Hbα (15 kDa) and Hbβ (16 kDa) were evidence that both represented monomers. Interestingly the larger molecular masses of spot 1 (27 kDa) and 2 (26 kDa) indicated the presence of Hbα complexes. Since exclusively Hbα was detected in these complexes, they are likely to represent homodimers. No corresponding changes in the Hbβ complexes could be seen due to a major overlapping of the spots, which is why it was difficult to characterize their possible composition.

### Decline of Hb complexes and monomers in the IPF but not in the COPD lungs

**Figure 2:**
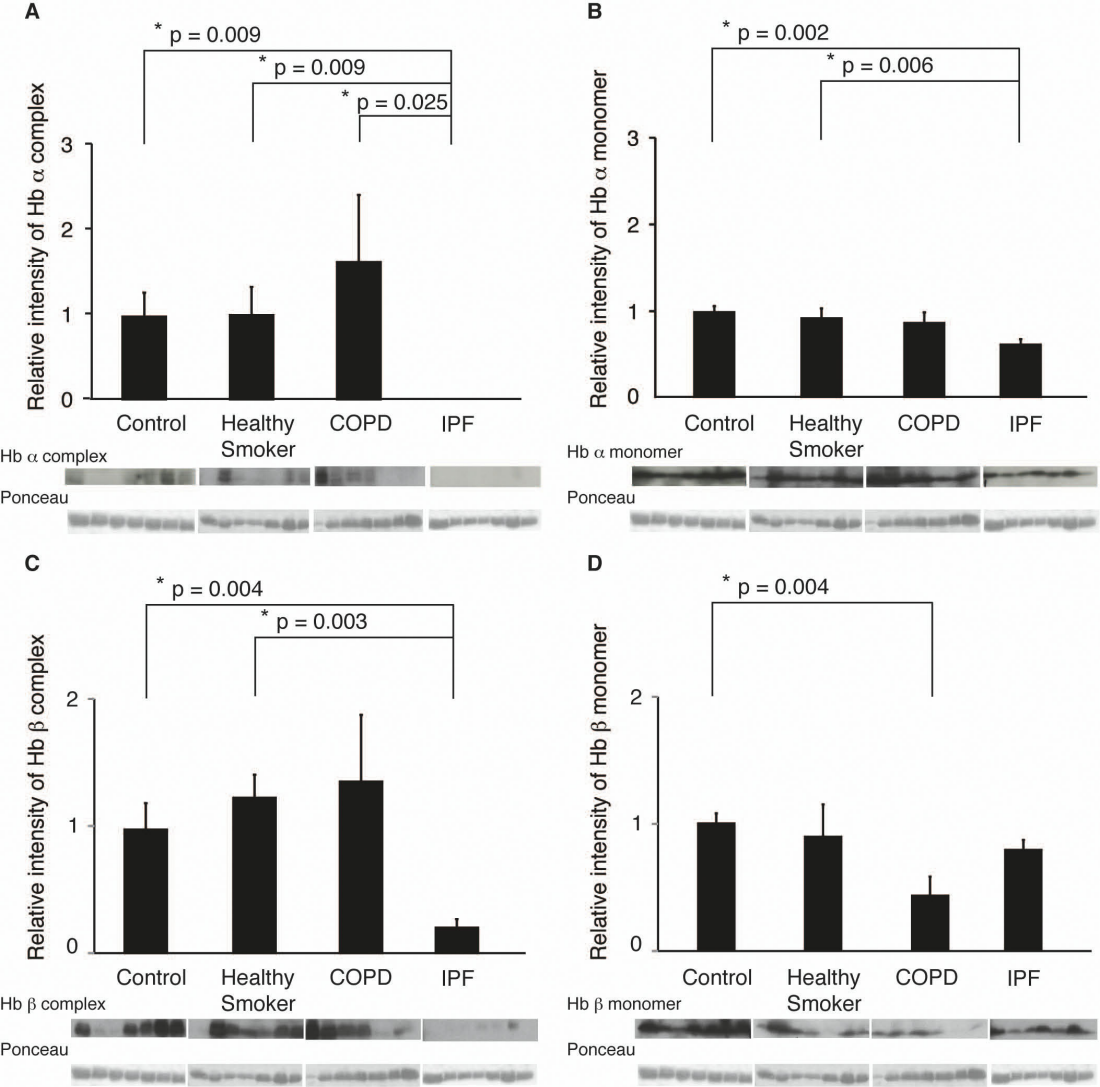
**Relative intensities of (A) Hbα complex and (B) monomer and (C) Hbβ complex and (D) monomer in control (n = 7), smoker (n = 7), COPD (n = 7) and IPF lungs (n = 7) determined by standard i.e. reducing Western blot analysis**. Data are presented as mean ± SEM. For patient characteristics see Table 2.

The Hbα and Hbβ levels were investigated next by Western blot using control lung (control; n = 7), IPF lungs (n = 7), and lung specimens from smokers without COPD (smokers; n = 7) and COPD (n = 7). Since Hb complexes, detected by 2-DE, are known to be formed through disulfide bonds [30], Hbα and Hbβ expression levels were evaluated in two ways i.e. reducing and non-reducing Western blot techniques. Western blot analyses confirmed the presence of the monomers and complexes of Hbα and Hbβ in the lung with corresponding molecular weights as in the 2DE. The results on the Hb complexes were very similar in the standard (Figure 2) and non-reducing Western blot (not shown) i.e. the presence of the Hbα complexes was completely missing or very low in the IPF lung. Also the levels of Hbα monomer were higher in the control than in the IPF lungs (1.6 fold) in the standard Western blot. In addition, the levels of Hbβ complexes were higher in the standard and non-reducing Western blots (4.6 and 5.1 fold) and the levels of Hbβ monomer higher (3.2 fold) in the non-reducing Western blots in the control than in the IPF lungs. The expression levels of Hbα, Hbβ or their complexes did not differ significantly in the lungs of the controls, smokers or patients with COPD except for the assays done under reducing conditions for Hbβ i.e. the level of Hbβ monomer was higher (2.3 fold) in the control than in the COPD lungs. These results in standard i.e. reducing Western blot conditions, are shown in Figure 2.

### Localization and quantification of Hbα and β immunoreactivity in the IPF and COPD lung

**Figure 3:**
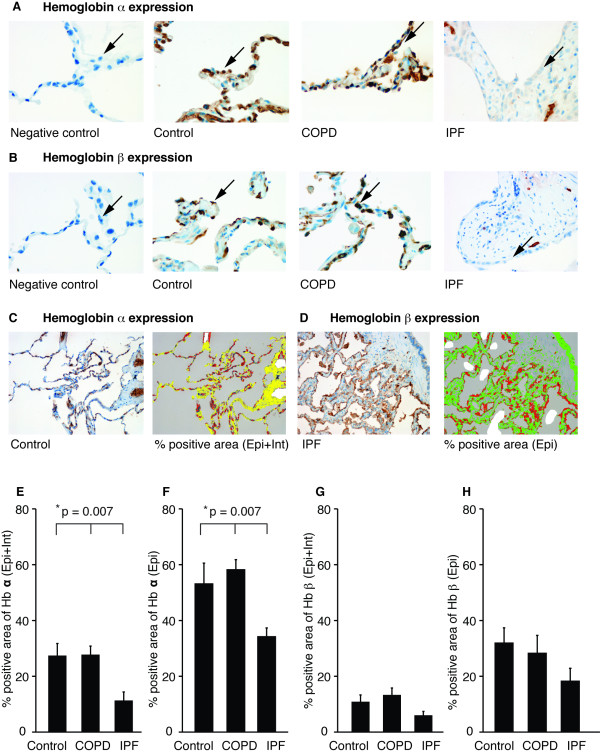
**Hbα and Hbβ expression and localization in representative sections in control, COPD and IPF lungs (A, B, magnification 900×)**. Positive Hbα and Hbβ expression was seen mainly in the alveolar epithelium as well as in macrophages in the control and COPD lungs. The alveolar epithelium (arrows) of patients with IPF displayed very weak staining in contrast to the situation in controls and patients with COPD. Both Hb stainings were low or absent in the fibrotic areas and fibroblast foci. Morphometrical analyses (magnifications 300×), which were conducted by excluding blood vessels and macrophages, show the sum of positive bronchiolar/alveolar epithelium and interstitium (Epi+Int) (C). Hbα and Hbβ positive area in bronchiolar/alveolar epithelium only was evaluated separately by excluding blood vessels, macrophages and interstitium (Epi) (D). Morphometrical analyses were evaluated from 6 control, 7 COPD and 7 IPF lung tissues. For detailed data see Additional files 1 and 2 (Tables S1 and S2; as shown in the Tables two or three representative areas were analyzed from all stained sections). Data are presented as mean ± SEM. For patient characteristics see Table 3.

The distribution of Hbα and Hbβ in the lung tissue was next investigated in control, IPF and COPD lung. In control and COPD lungs, Hbα and Hbβ could be clearly detected in the alveolar epithelium with some positivity of Hbα being found also in the bronchiolar/bronchial epithelium and macrophages (Figure 3A, 3B, high magnification). Both Hbα and Hbβ immunoreactivities were low or absent in the alveolar regions, interstitium and fibroblast foci in the IPF lung. Morphometrical analysis, which was conducted by excluding blood vessels and macrophages (since they contain erythrocytes and Hb), shows the sum of positive bronchiolar/alveolar epithelium and interstitium; Epi+Int) (Figure 3C). In addition, the Hbα and Hbβ positive areas in bronchiolar/alveolar epithelium were evaluated by excluding blood vessels, macrophages and interstitium (Epi) (Figure 3D). Next, the positive area of Hbα and Hbβ was calculated by using morphometry. As shown in Figure 3E and 3F, the Hbα positive areas (Epi+Int and Epi) between the control, COPD and IPF groups differed significantly (Kruskal Wallis test; *p *= 0.007) while the Hbβ positive areas (Epi+Int and Epi) did not (Figure 3G, 3H).

### Prevention of Hbα complex formation by cysteine 105 modification in the IPF lung

**Figure 4:**
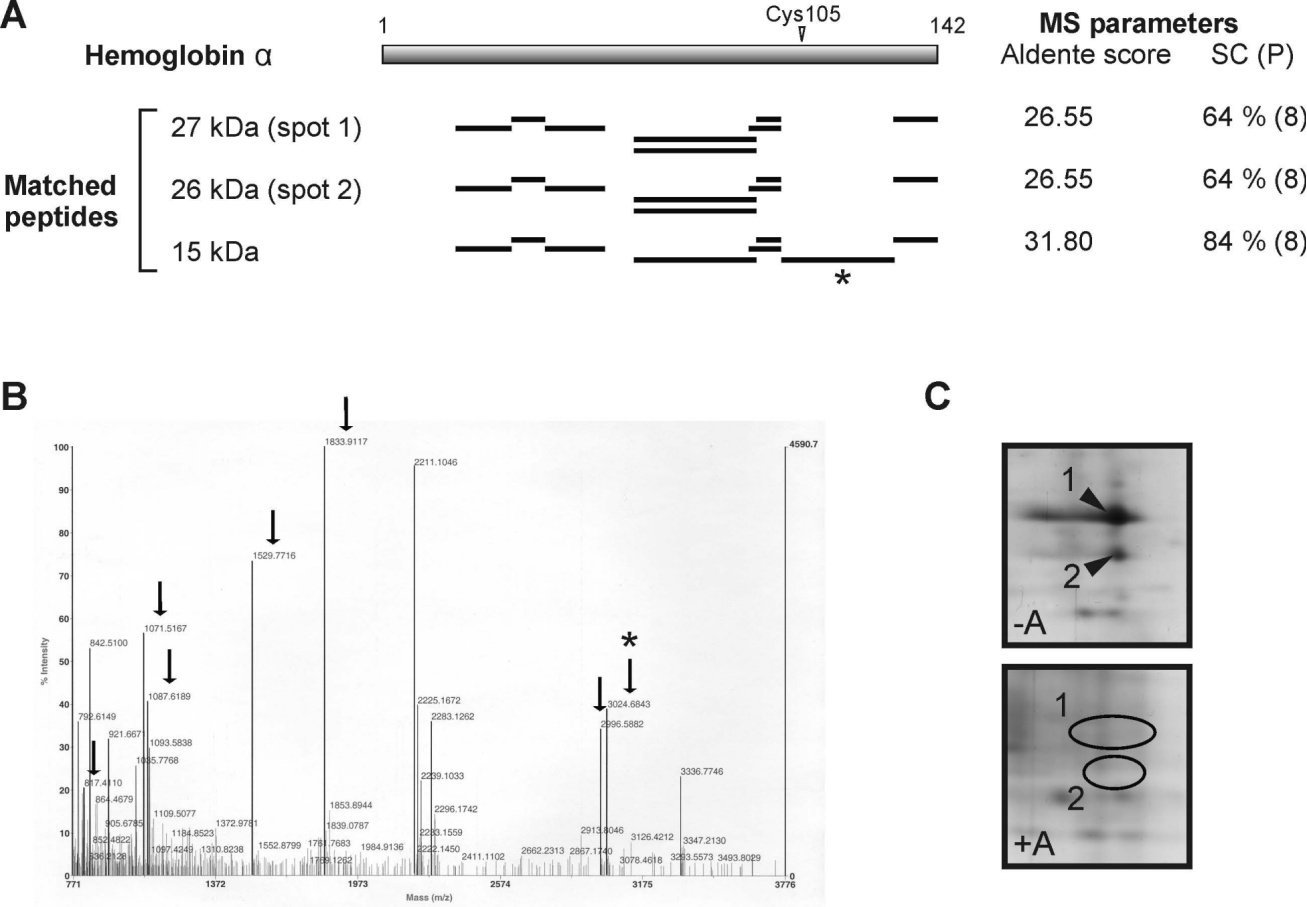
**Modification at cysteine 105 prevents formation of Hbα complexes**. (A) Schematic presentation of the spot-specific peptides, obtained by MS, and the covered protein sequence. An asterisk indicates the cysteine-containing peptide 3024.6338. MS parameters represent Aldente score, sequence coverage (SC) and the number of matched peptides (P). (B) MS spectrum representative for the Hbα monomer. (C) Gel parts corresponding to spots marked in Figure 1 revealed the presence of Hbα complexes without (-A) and with (+A) alkylation prior to separation. Homogenates of control lungs were separated by 2-DE (pH 6-11) and the total protein pattern visualized by silver staining.

In the IPF lungs, only a modest reduction of the Hbα monomer level was observed whereas the Hbα complex was absent (Figures 1, 2). This hinted that an additional mechanism might be effecting the complex formation. It has been shown that Hb complexes, detected by 2-DE of purified human globin chains, are formed through disulfide bonds [30]. Hbα contains only one cysteine at position 105 (Cysα105) likely to be the site responsible for complex formation. In agreement, MS analyses revealed that the peptide 3024.6338 containing Cysα105 was present in the major Hbα spots at 15 kDa but not in the Hbα complexes (Figure 4A, 4B). Therefore the possibility of complex formation through this cysteine was investigated in more detail. Alkylation prior to separation abolished the presence of Hbα at the higher molecular mass, indicating that Cysα105 was indeed involved in the complex formation (Figure 4C). Overall, the reduced levels of the Hbα complexes in the IPF lungs point to the presence of a modified thiol group at Cysα105 preventing the complex formation. It is very likely that the oxidative stress in the IPF lungs results in the redox regulated modification at Cysα105, e.g. S-glutathiolation, S-nitrosylation or formation of sulfonic acid. Moreover, it is possible that thiol groups in both Hb subunits may be nitrosylated or nitrated *in vivo*, since corresponding findings have been documented to occur also with Hbβ [31,32].

### Occurrence of Oxidative/Nitrosative Stress in the IPF and COPD lung

**Figure 5:**
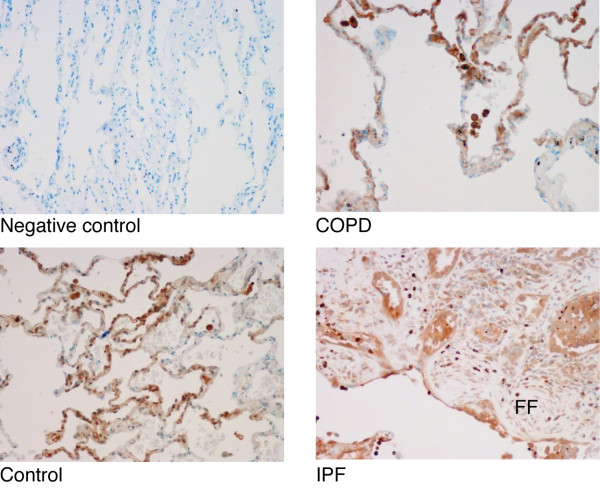
**Nitrotyrosine expression and localization in representative sections of negative control, control, COPD and IPF lungs**. Positive nitrotyrosine expression is seen mainly in the epithelial cells and inflammatory cells in both diseases but not in the fibrotic lesions or fibroblast foci in the IPF lung. There is some nitrotyrosine positivity also in the control (ex-smoker) lung. For patient characteristics see Table 3.

Due to the disturbance of the Hbα complex formation, most likely via nitrosylation or nitration, only in IPF but not in COPD lung, it was decided to investigate whether these two diseases, IPF and COPD, display any major differences in nitrotyrosine expression in general. Our earlier studies have revealed that there is remarkable nitrotyrosine positivity, especially in the fibrotic lung [22,29], while another study from our laboratory showed relatively weak nitrotyrosine expression in the COPD lung parenchyma [33]. This comparison was conducted by staining both the IPF and COPD lung tissues with the same techniques at the same time and by analyzing the positivity in a semiquantitative manner by Western blot analysis. The results showed clear nitrotyrosine positivity, especially in the epithelial cells and inflammatory cells but not in the interstitium or fibroblast foci in IPF. In COPD, nitrotyrosine positivity was especially localized in the epithelium and inflammatory cells (Figure 5). In addition, the control lung showed nitrotyrosine positivity with possible reasons including anesthesia, ventilation with high oxygen and the generated stress reaction during lung surgery. Western blot showed extensive nitrotyrosine positivity, but when normalized against the loaded protein in the lane, no major difference between these diseases could be seen (not shown). However, if the total nitrotyrosine immunoreactivity in the lung parenchyma of the inflated IPF and COPD lung is calculated against the surface area, the values in the COPD lung remain low which is then related only to the large emphysematous areas in the COPD lung with no tissue/alveoli. It remains unclear if these kinds of differences can contribute to the oxidant burden in the IPF or COPD lung *in vivo*.

### Hb expression as tetramers in BALF and induced sputum samples

**Figure 6:**
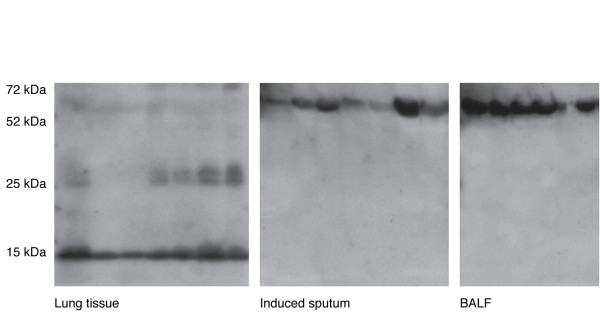
**The expression of Hb by standard Western blot analysis in the lung homogenates (n = 6), BALF (n = 6) and induced sputum supernatants (n = 7) of control subjects**. BALF had been obtained from subjects with prolonged cough who had normal spirometry, BAL cell profile and HRCT finding. Induced sputum was obtained from healthy non-smokers. The results indicate that the major Hb forms detectable by the commercial antibodies differ between the lung tissue and BALF or sputum, the major band in the lung tissue being the Hbα and Hbβ (not shown in this figure) complex, while it is detected as larger complexes corresponding to Hb tetramers in the "secretions". The expression by using the Hbα and β antibodies in the BALF and sputum was very similar further suggesting that the band represents tetramer. Here only Hbα is shown. For patient characteristics see Table 4.

The secretion of Hbα and Hbβ into BALF and induced sputum supernatant was investigated in subjects with normal lung function values to determine whether Hb could be detected in these specimens. Furthermore, the secreted forms were compared to those in the lung tissue homogenates. The Hb forms differed between the lung homogenates and BALF or sputum supernatants, the major bands in the lung tissue consisting of the Hbα and Hbβ complexes and monomers, while the major band in the "secretions" corresponded to the molecular weight of Hb tetramer (Figure 6). The bands were similar in the BALF and sputum supernatants as confirmed with the Hbα and Hbß antibodies i.e there was the presence of complexes containing both Hbα and Hbß i.e. tetramers in both secretions. Hb complexes or monomers could barely be detected in the BALF or sputum supernatants by Western analysis. It was not possible to determine whether Hb levels vary between the controls and the disease states since the major difference i.e. the changes in the Hbα complexes and also Hb monomers in the tissues, were not clearly detectable in the secretions.

## Discussion

In the present study, unbiased proteomics and subsequent MS and Western blot analyses indicated reduced levels of Hb (α, β) monomers and complexes in lung specimens from patients with IPF compared to the controls. According to the immunohistochemistry, normal human lung expressed Hbα and Hbβ most prominently in the alveolar epithelial cells while in the IPF lung, the levels of both Hb monomers were very low or even undetectable. Subsequent studies (2-DE, Western blot, immunohistochemistry, morphometry) on COPD, a disease with a different type of parenchymal lung damage, detected no or very minimal changes in the expression of Hbα and Hbβ compared to control with both Hb forms being localized mainly in the alveolar epithelium of COPD lungs. A detailed MS-analysis indicated that a disturbance in the complex formation of Hbα in the IPF lung was associated with the modification of a thiol group (Cysα105) present in the Hbα molecule. Additional studies on BALF samples and induced sputum supernatants revealed that Hb could be detected in these specimens mainly as tetramers.

There are several problems in proteomic studies which are related to the sample type in various parenchymal lung diseases as reviewed in [34]. In the present study, we examined lung tissue specimens in our proteomic analyses from two different types of parenchymal lung diseases i.e. IPF and COPD to obtain a broad perspective of the overall lung pathology. To avoid the problems and overlapping features of these diseases, IPF cases were selected from never or ex-smokers with short smoking histories, and COPD cases represented end stage disease with severe emphysema. These findings suggest that the changes in the Hb monomers and/or complexes may be related to a specific type of parenchymal lung damage.

The human Hb molecules are a set of very closely related proteins formed by symmetric pairing of a dimer of polypeptide chains, the α- and β-globins, into a tetrameric structural and functional unit. The α_2_β_2 _molecule represents the predominant adult Hb [35]. Originally detected in erythroid cells, Hb expression has been detected in eye, kidney, endometrium, activated macrophages and cultured alveolar epithelial cells [36-43]. Our immunoblotting technique identified not only the Hbα and Hbβ monomers but also their complexes in human lungs, whereas decline in IPF was most significant in the Hbα complex. The positive immunoreactivity of the Hb monomers in alveolar macrophages may be partly related to red blood cell phagocytosis in the diseased lung. In contrast, the expression of the Hb monomers in the alveolar epithelial cells is in full agreement with previous findings on the airway epithelium [39].

The distributions of Hbα and Hbβ were evaluated in human lung tissues by immunohistochemistry and their expression by morphometry of areas which did not include blood vessels or macrophages. Hbα and Hbβ were mainly localized in alveolar cells. On the other hand, the alveolar epithelium of patients with IPF displayed weaker staining in contrast to the controls, smokers and patients with COPD. Interestingly, lung lavage samples of smokers and COPD patients have been shown to exhibit elevated concentrations of both iron and ferritin compared to healthy non-smokers, suggesting that cigarette smoke exposure can alter iron homeostasis in the lung [44]. It is not known whether these changes impact on the expression Hb in the COPD lung, although some kind of association is not impossible. In agreement, immunohistochemistry of the COPD lung revealed an intensively stained alveolar region containing the Hb units. The situation is different in IPF where the alveolar epithelium is replaced by a thick fibrotic barrier against diffusion. Overall, these results suggest that the two Hb monomers, Hbα and Hbβ may play important, but different roles in the pathogenesis of IPF and COPD.

The studies were extended to BALF and induced sputum supernatants, since bronchofiberoscopy and BAL are widely used in the differential diagnosis of IPF and induced sputum reflects the airway inflammation/pathology in chronic airway diseases. There is one study that has evaluated Hb monomers from sputum by SELDI-MS but this approach was focused on single proteins, not protein complexes [45]. Our studies using Western blot and commercial antibodies indicate that Hb is secreted to these samples and is present mainly as the larger complexes containing both Hbα and Hbβ corresponding to Hb tetramers. Since no complexes or monomers could clearly be detected from these samples, more sensitive and still unavailable methods will need to be developed before this hypothesis can be tested. Interestingly even concentrated BALF samples were negative for Hbα complexes, this representing a major difference between the control and IPF lung by 2DE, Western blot and morphometry in the lung tissue specimens. These preliminary findings and their significance need to be confirmed in future investigations.

The main function of Hb is to transport oxygen from lung to tissues, and lung is very sensitive to changes in oxygen delivery [35]. Hb represents a highly reactive molecule which has, in addition to its oxygen-carrying capacity, a multitude of enzymatic, protective, NO neutralizing and ligand binding activities [46]. Protein *S*-nitrosylation of Cys residues also accounts for a large part of the ubiquitous influence of NO on the cellular signal transduction pathway [31,32], and the interactions between NO and the Hb monomers have been shown to regulate physiological responses such as vasodilatation and vasoconstriction [47]. The detection of corresponding alterations in Hbα but not in Hbβ complexes by 2-DE in the present study might be explained by the overlapping of Hbβ complexes with other proteins which allowed no reliable analysis. In fact, several investigators have emphasized the importance of Hbβ nitrosylation/denitrosylation reactions in the pathologies of many diseases *in vivo *[14]. Overall, this suggests that a similar modification not only for Hbα but also for Hbβ may occur simultaneously in the IPF lungs.

The levels of Hb complexes declined only in IPF for reasons that remain unclear. The prevention of the complex formation was investigated using both standard and non-reducing Western blot, the results were similar. It is possible that our reducing conditions did not cause total reduction, especially of the highly abundant proteins, in the specimens. One could also speculate that the handling of the tissues may have caused some of the changes, though all tissues underwent similar processing. This study included relatively low numbers of the patients with IPF and COPD. Nonetheless, the results were very clear, and the changes between the controls and IPF were not only very significant but also could be confirmed by many methods. In addition, this study is the first to characterize the Hb in human lung and lung diseases. The changes in the Hb composition could be seen not only in the complex formation but also in both Hb monomers. Instead, Hb was detected in the secretions such as BALF and induced sputum mainly as high molecular complexes corresponding to Hb tetramers. Currently, no commercial ELISA is available for the detection of different Hb variants in BALF or sputum to allow the evaluations of their exact changes in various clinical conditions. An understanding of the exact mechanism and significance of the decline and modification of Hb units in IPF but not in COPD will demand further studies both in experimental models of lung fibrosis and COPD.

## Conclusions

This is the first study showing the expression of Hb in human lung and there mainly in alveolar epithelium. In IPF, Hb complex formation is prevented. These results can be considered to have widespread implications also in several other chronic inflammatory diseases where oxygen transport/saturation and exchange are disturbed.

## List Of Abbreviations

IPF: idiopathic pulmonary fibrosis; COPD: chronic obstructive pulmonary disease; Hb: hemoglobin; HRCT: high resolution computed tomography; MS: mass spectrometry; UIP: usual interstitial pneumonia; BALF: bronchoalveolar lavage fluid; 2-DE: two-dimensional gel electrophoresis.

## Competing interests

The authors declare that they have no competing interests.

## Authors' contributions

NI participated in the design of the study, analyzed the Western and immunohistochemical results, performed part of the statistical analysis and drafted the manuscript. SO carried out the proteomic analysis and participated in creating the figures. KS participated in the analyzing immunohistochemical results. MM participated in the design of the study and collection of patient material. IR participated in the design of the study. WM participated in selection and collection of patient material, analyzing the Western analysis results and performed part of the statistical analysis and participated in creating the figures. VLK conceived the study, and participated in its design and coordination and helped to draft the manuscript. All authors have read and approved the final manuscript.

## Supplementary Material

Additional file 1 **Table S1**. Detailed data of the morphometrical analysis of Hbα and Hbβ positive area (sum of the bronchial/alveolar epithelium and interstitium; Epi+Int) Click here for file

Additional file 2 **Table S2**. Detailed data of the morphometrical analysis of Hbα and Hbβ positive area (sum of the bronchial/alveolar epithelium; Epi) Click here for file
