# Supplementary material for: Hemoglobin α and β are ubiquitous in the human lung, decline in idiopathic pulmonary fibrosis but not in COPD
Source: Respir Res. 2010 Sep 13;11(1):123. doi: 10.1186/1465-9921-11-123 (PMC2949726; doi:10.1186/1465-9921-11-123)
Supplement: Additional file 1 — Table S1. Detailed data of the morphometrical analysis of Hbα and Hbβ positive area (sum of the bronchial/alveolar epithelium and interstitium; Epi+Int) [file 1465-9921-11-123-S1.DOC]

**Table S1**. Detailed data of the morphometrical analysis of Hbα and Hbβ positive area (sum of the bronchial/alveolar epithelium and interstitium; Epi+Int)

| Case | Hbα | | | |  | Hbβ | | | |
| --- | --- | --- | --- | --- | --- | --- | --- | --- | --- |
|  | Area1 | Area2 | Area3 | Mean |  | Area1 | Area2 | Area3 | Mean |
| Control, Case1 | 13.7 | 15.6 |  | 14.6 |  | 4.9 | 3.2 |  | 4.0 |
| Control, Case 2 | 18.3 | 41.0 |  | 29.7 |  | 12.1 | 12.9 |  | 12.5 |
| Control, Case 3 | 39.0 | 28.1 |  | 33.5 |  | 16.9 | 16.7 |  | 16.8 |
| Control, Case 4 | 25.5 | 11.1 |  | 18.3 |  | 5.0 | 1.5 |  | 3.3 |
| Control, Case 5 | 65.4 | 22.2 |  | 43.8 |  | 9.6 | 16.7 |  | 13.1 |
| Control, Case 6 | 19.6 | 29.5 |  | 24.6 |  | 18.7 | 14.4 |  | 16.6 |
| COPD, Case 1 | 27.8 | 32.4 |  | 30.1 |  | 34.1 | 17.5 |  | 25.8 |
| COPD, Case 2 | 14.7 | 26.9 | 21.5 | 21.5 |  | 4.4 | 4.6 |  | 4.5 |
| COPD, Case 3 | 18.6 | 31.3 | 29.1 | 29.1 |  | 14.6 | 6.5 |  | 10.5 |
| COPD, Case 4 | 58.9 | 23.6 |  | 41.2 |  | 14.7 | 9.3 |  | 12.0 |
| COPD, Case 5 | 24.9 | 32.5 | 32.5 | 32.5 |  | 15.6 | 16.1 |  | 15.8 |
| COPD, Case 6 | 8.6 | 42.0 | 37.5 | 37.5 |  | 7.6 | 14.0 |  | 10.8 |
| COPD, Case 7 | 22.0 | 10.8 |  | 16.4 |  | 17.3 | 13.0 |  | 15.2 |
| IPF, Case 1 | 1.2 | 2.6 | 3.3 | 2.6 |  | 3.5 | 6.8 | 5.2 | 5.2 |
| IPF, Case 2 | 2.3 | 2.2 | 6.5 | 2.3 |  | 4.0 | 1.3 | 1.0 | 2.1 |
| IPF, Case 3 | 3.9 | 10.0 | 15.4 | 10.0 |  | 9.3 | 2.9 | 18.5 | 10.2 |
| IPF, Case 4 | 21.8 | 36.1 | 22.7 | 22.7 |  | 7.1 | 4.8 | 9.9 | 7.3 |
| IPF, Case 5 | 15.5 | 8.0 |  | 11.8 |  | 1.2 | 1.2 |  | 1.2 |
| IPF, Case 6 | 6.3 | 17.2 | 18.1 | 17.2 |  | 11.3 | 5.8 | 10.7 | 9.3 |
| IPF, Case 7 | 7.6 | 9.7 | 15.8 | 9.7 |  | 0.3 | 3.8 | 20.2 | 8.1 |
