# Supplementary material for: Hemoglobin α and β are ubiquitous in the human lung, decline in idiopathic pulmonary fibrosis but not in COPD
Source: Respir Res. 2010 Sep 13;11(1):123. doi: 10.1186/1465-9921-11-123 (PMC2949726; doi:10.1186/1465-9921-11-123)
Supplement: Additional file 2 — Table S2. Detailed data of the morphometrical analysis of Hbα and Hbβ positive area (sum of the bronchial/alveolar epithelium; Epi) [file 1465-9921-11-123-S2.DOC]

**Table S2**. Detailed data of the morphometrical analysis of Hbα and Hbβ positive area (sum of the bronchial/alveolar epithelium; Epi)

| Case | Hbα | | | |  | Hbβ | | | |
| --- | --- | --- | --- | --- | --- | --- | --- | --- | --- |
|  | Area1 | Area2 | Area3 | Mean |  | Area1 | Area2 | Area3 | Mean |
| Control, Case1 | 41.3 | 50.7 |  | 46.0 |  | 48.9 | 15.1 |  | 32.0 |
| Control, Case 2 | 75.0 | 71.7 | 87.4 | 78.0 |  | 14.4 | 44.8 |  | 29.6 |
| Control, Case 3 | 58.7 | 44.1 |  | 51.4 |  | 17.8 | 71.5 |  | 44.6 |
| Control, Case 4 | 32.1 | 32.2 |  | 32.2 |  | 15.9 | 9.3 |  | 12.6 |
| Control, Case 5 | 87.1 | 53.3 |  | 70.2 |  | 45.1 | 50.3 |  | 47.7 |
| Control, Case 6 | 39.8 | 42.6 |  | 41.2 |  | 25.5 | 26.9 |  | 26.2 |
| COPD, Case 1 | 87.4 | 30.8 |  | 59.1 |  | 72.6 | 43.4 |  | 58.0 |
| COPD, Case 2 | 37.4 | 48.8 | 50.3 | 45.5 |  | 26.5 | 7.1 |  | 16.8 |
| COPD, Case 3 | 46.9 | 46.2 | 72.1 | 55.0 |  | 48.0 | 33.9 |  | 41.0 |
| COPD, Case 4 | 36.8 | 66.8 |  | 51.8 |  | 20.0 | 44.5 |  | 32.3 |
| COPD, Case 5 | 76.9 | 77.7 | 54.3 | 69.6 |  | 22.4 | 21.4 |  | 21.9 |
| COPD, Case 6 | 35.0 | 58.9 | 75.3 | 56.4 |  | 14.1 | 15.0 |  | 14.6 |
| COPD, Case 7 | 83.4 | 56.5 |  | 69.9 |  | 14.7 | 14.6 |  | 14.6 |
| IPF, Case 1 | 29.7 | 52.5 | 1.0 | 27.7 |  | 43.2 | 20.9 | 6.2 | 23.4 |
| IPF, Case 2 | 7.2 | 10.5 | 75.9 | 31.2 |  | 4.5 | 9.3 | 7.5 | 7.1 |
| IPF, Case 3 | 18.6 | 32.2 | 58.6 | 36.4 |  | 21.5 | 15.0 | 58.7 | 31.7 |
| IPF, Case 4 | 27.1 | 58.4 | 65.5 | 50.3 |  | 15.8 | 67.0 | 7.4 | 30.1 |
| IPF, Case 5 | 15.8 | 54.2 |  | 35.0 |  | 3.5 | 1.4 | 0.0 | 1.6 |
| IPF, Case 6 | 3.3 | 43.5 | 36.0 | 27.6 |  | 11.9 | 7.5 | 52.7 | 24.0 |
| IPF, Case 7 | 27.8 | 49.2 | 19.5 | 32.2 |  | 3.9 | 14.3 | 13.8 | 10.7 |
